# Separation of Rare-Earth Elements from Nitrate Solutions by Solvent Extraction Using Mixtures of Methyltri-n-octylammonium Nitrate and Tri-n-butyl Phosphate

**DOI:** 10.3390/molecules27020557

**Published:** 2022-01-16

**Authors:** Sergei I. Stepanov, Nguyen Thi Yen Hoa, Ekaterina V. Boyarintseva, Alexander V. Boyarintsev, Galina V. Kostikova, Aslan Yu. Tsivadze

**Affiliations:** 1 Department of Technology of Rare Elements and Nanomaterials Based on Them, Mendeleev University of Chemical Technology, 9 Miusskaya Square, 125047 Moscow, Russia; sstepanov@muctr.ru (S.I.S.); yenhoa7234@gmail.com (N.T.Y.H.); eboyarinceva@muctr.ru (E.V.B.); 2 A.N. Frumkin Institute of Physical Chemistry and Electrochemistry, Russian Academy of Sciences, 31/4 Leninsky Prospect, 119071 Moscow, Russia; galyna_k@mail.ru (G.V.K.); tsiv@phyche.ac.ru (A.Y.T.)

**Keywords:** solvent extraction, rare-earth elements, methyltrioctylammonium nitrate, tri-n-butyl phosphate, synergic mixture, nitrate solutions, lanthanum, cerium, praseodymium, neodymium

## Abstract

The article presents data on the solvent extraction separation of rare-earth elements (REEs), such as La(III), Ce(III), Pr(III), and Nd(III), using synergic mixtures of methyltrioctylammonium nitrate (TOMANO_3_) with tri-n-butyl phosphate (TBP) from weakly acidic nitrate solutions. Specifically, experimental results on separation of REEs, for the pair Ce(III)/Pr(III) for quaternary mixtures of REEs (La(III), Ce(III), Pr(III), Nd(III)) and for the pair La(III)/Pr(III) for solutions containing La(III), Pr(III), and Nd(III), are presented. It was shown that effective separation for the pair Ce(III)/Pr(III) from a solution containing 219 g Ce(III)/L, 106 g La(III)/L, 20 g Pr(III)/L, 55 g Nd(III)/L, and 0.1 mol/L HNO_3_, was achieved using 56 steps of a multistage, counter-current solvent extraction cascade with scrubbing, at an organic-to-aqueous phase volume ratio (O/A) equal to 2/1 on the extraction section and O/A equal to 4/1 on the scrubbing section, using 3.3 mol/L solutions of the mixture TOMANO_3_-TBP with molar ratio 0.15:0.85 in dodecane. Separation for the pair La(III)/Pr(III) could be achieved using a solvent extraction cascade with scrubbing in 32 steps at O/A equal to 2/1 on the extraction section and O/A equal to 2.8/1 on the scrubbing section of the solvent extraction cascade from a solution containing 258 g La(III)/L, 58 g Pr(III)/L, 141 g Nd(III)/L, and 0.1 mol/L HNO_3_ with 3.0 mol/L solution of the mixture TOMANO_3_-TBP with molar ratio 0.2:0.8 in dodecane.

## 1. Introduction

Rare earth elements (REEs) are most often used in nuclear technology, ferrous and non-ferrous metallurgy, electronics and radio engineering, the chemical and silicate industry, medicine, and agriculture. The REE group contains 17 elements, including scandium (Sc), yttrium (Y), lanthanum (La), and the lanthanides (Ln): cerium (Ce), praseodymium (Pr), neodymium (Nd), samarium (Sm), europium (Eu), gadolinium (Gd), terbium (Tb), dysprosium (Dy), holmium (Ho), erbium (Er), thulium (Tu), ytterbium (Yb), and lutetium (Lu).

In medicine, REEs are applied in the fabrication process of medication for the clinical management of various tumors, tuberculosis, leprosy, eczema, gout, rheumatism, and gastric diseases. REEs help against marine disease and chronic vomiting syndrome (e.g., cerium salts) and are also components of embalming compositions. Organic salts of REEs have antiseptic effects (e.g., salicylates of neodymium and praseodymium) and are also used as anticoagulants. Gadolinium chloride is used to block Kupffer cells in the treatment of the liver. Some REE salts prevent blood clotting, making them valuable for blood preservation. Erbium and cerium salts increase the content of hemoglobin and red blood cells. In medical equipment, dysprosium is applied in medical lasers. Cerium and scandium are used for the manufacture of dentures [1].

In nuclear and radiation medicine, isotopes ^177^Lu, ^46^Sc are used for the treatment of cancer tumors, isotope ^153^Gd for diagnosis of osteoporosis, and isotope ^170^Tm for the manufacture of portable X-ray medical installations.

The use of REE compounds in agriculture as microfertilizers and insectofungicides is increasing. REEs can also increase the yield of crops (e.g., rice, wheat, corn, sugarcane, sugar beets, tobacco, tea, cotton, peanuts, fruits, flowers, and others) [1].

It is necessary to develop new, or to modernize current, schemes and processes, particularly new efficient and safer solvent extraction (SE) systems for the separation of multicomponent solutions, to improve reprocessing technology for mineral and technogenic REE-containing deposits. SE separation of REEs from nitrate or chloride aqueous solutions is widely used in global practice to produce pure individual metals and compounds [2]. As initial raw materials, collective REE concentrates obtained from various ores are used. Usually, the separation of collective concentrates is carried out into three groups, such as light (La, Ce, Pr, Nd), medium (Sm, Eu, Gd), and heavy (Tb, Dy, Ho, Er, Tu, Yb, Lu), followed by the recovery of individual REEs. Generally, all SE separation processes operate using selective solvents of various classes or their synergic mixtures [3].

Tri-n-butyl phosphate (TBP) is widely used in practical applications to separate light REEs from middle and heavy REEs and to separate these into individual elements. Organophosphorus and carbonic acids, such as di-(2-ethylhexyl)phosphoric acid (DEHPA or HDEHP), isododecylphosphetanic acid (IDPA), di-(2,2,4-trimethylpenthyl)phosphinic acid (Cyanex 272), 2-ethylhexyl phosphonic acid mono-2-ethylhexyl ester (PC-88A), and α-branched tertiary carboxylic acids (C_8_–C_16_ higher isomeric acids and neodecanoic—Versatic 10 acid) became widely used in industrial practice for separation of middle and heavy REEs [3]. For yttrium recovery from heavy REE concentrates, the use of tertiary and quaternary ammonium compounds (QACs), in particular, tri-n-alkylammonium nitrate (TAA), methyltri-n-alkyl(octyl)ammonium nitrate (TAMA(TOMA)NO_3_) and analogues including Aliquat 336, N_263_(methyltri-n-octylammonium chloride), Adogen 464 (methyltrialkyl(C_8_–C_10_)ammonium chloride) [4] has been proposed.

Among various solvent mixtures, including synergic ones, solutions of TAMANO_3_-TBP mixtures in hydrocarbon diluents for separation of middle REEs, including the pair Sm(III)/Gd(III), following chemical recovery of europium from nitrate solutions, became widely used in the industry [5]. TAMANO_3_ (TOMANO_3_)-TBP mixtures have a significant synergistic effect during SE of various REEs from nitrate solutions which increases both the distribution ratios of individual elements (D_Ln_), and the separation factors of REE pairs (β_Ln1/Ln2_).

The synergistic properties of TAMANO_3_ (TOMANO_3_)-TBP mixtures are due to the formation of mixed complex compounds in the organic phase between the extracted metal and two solvents. The composition of the extracted REE compounds in systems with TAMANO_3_ (TOMANO_3_)-TBP differs for different individual elements and depends on the Ln(III)-TAMANO_3_-TBP ratios, the concentration of lanthanide, and each of the solvents of the mixture. In early work on the chemistry of extraction of lanthanides of the light group, as a rule, methods were used to determine the composition of the extracted compound allowing identification of only one of the extracted compounds, due to the limitations of the techniques used. Table 1 presents generalized data on the compositions of the extracted compounds. These data do not reflect the entire range of solvent extraction chemistry due to changes in the conditions of its implementation and, particularly, changes in the molar ratios of Ln(III)-TAMANO_3_-TBP.

S.I. Stepanov et al. applied an approach that allows definition of the variety of extractable compounds in a wide range of a mixture’s molar ratios of lanthanide and solvents [15,16,17]. In studies [16,17], the SE chemistry of La(III), Ce(III), Pr(III), and Nd(III) using TOMANO_3_-TBP mixtures was investigated applying a wide range of changes in their molar ratios in toluene solutions using the isomolar series method. By studying the SE of these four REEs using TOMANO_3_-TBP mixtures, the manifestation of a synergistic effect (S_Ln_) was established, and the areas of synergistic SE and the compositions of extractable mixed complexes in maxima on the curves S_Ln_ = *f*(composition of the mixture) were determined.

The SE of La(III) from a solution containing 0.88 mol/L La(NO_3_)_3_, 4.0 mol/L NH_4_NO_3_, and 0.01 mol/L HNO_3_ using 1.0 mol/L isomolar mixtures of TOMANO_3_-TBP in toluene was accompanied by the manifestation on the synergetic curve of two maxima corresponding to the formation of the complexes (R_4_N)[La(NO_3_)_4_·(2–3)TBP] and (R_4_N)_2_[La(NO_3_)_5_·2TBP] for synergetic compositions 0.15 mol/L TOMANO_3_-0.85 mol/L TBP and 0.9 mol/L TOMANO_3_-0.1 mol/L TBP [16].

The SE of Ce(III), using 1.0 mol/L TOMANO_3_-TBP mixtures in toluene from solutions containing 1.0 mol/L Ce(NO_3_)_3_, 4.0 mol/L NH_4_NO_3_, and 0.01 mol/L HNO_3_, was accompanied by the consecutive formation of complexes with the following compositions: R_4_N[Ce(NO_3_)_4_·4TBP], (R_4_N)_2_[Ce(NO_3_)_5_·3TBP], (R_4_N)_2_[Ce(NO_3_)_5_·2TBP], (R_4_N)_3_[Ce(NO_3_)_6_·TBP], and (R_4_N)_3_[Ce(NO_3_)_6_]. The synergic SE domain ranged from the composition 0.2 mol/L TOMANO_3_-0.8 mol/L TBP to 0.7 mol/L TOMANO_3_-0.3 mol/L TBP [16].

For Pr(III), during SE from a solution containing 0.487 mol/L Pr(NO_3_)_3_, 4.0 mol/L NH_4_NO_3_, 0.01 mol/L HNO_3_, 1.0 mol/L using TOMANO_3_-TBP mixtures in toluene, the synergic curve featured four maxima in TOMANO_3_ concentration changes ranging from 0.2 mol/L to 0.65 mol/L. These maxima are consistent with sequential formation (with growing TOMANO_3_ concentration) of the Pr(III) mixed complexes, R_4_N[Pr(NO_3_)_4_·4TBP], R_4_N[Pr(NO_3_)_4_·3TBP], R_4_N[Pr(NO_3_)_4_·2TBP], (R_4_N)_2_[Pr(NO_3_)_5_·3TBP], (R_4_N)_2_[Pr(NO_3_)_5_·2TBP] and (R_4_N)_3_[Pr(NO_3_)_6_·2TBP], which confirms the mechanism of substitution of the phosphoryl ligand of TBP molecules by NO_3_ ligands of TOMANO_3_ [17].

During SE of Nd(III), from a solution containing 0.429 mol/L Nd(NO_3_)_3_, 4.0 mol/L NH_4_NO_3_, 0.01 mol/L HNO_3_ using 1.0 mol/L isomolar TOMANO_3_-TBP mixtures in toluene, the synergic curve fully fitted in the area S_Nd(III)_ > 1 which indicates the occurrence of synergic SE for all mixture compositions studied. Four maxima, ranging from 0.2 mol/L TOMANO_3_-0.8 mol/L TBP to 0.85 mol/L TOMANO_3_-0.15 mol/L TBP corresponded to the sequential formation of the following compounds: R_4_N[Nd(NO_3_)_4_·4TBP], (R_4_N)_2_[Nd(NO_3_)_5_·3TBP], (R_4_N)_3_[Nd(NO_3_)_6_·2TBP], and (R_4_N)_4_[Nd(NO_3_)_7_·TBP]. The latter complex can be considered a three-charge mixed complex (R_4_N)_3_[Nd(NO_3_)_6_·TBP]·R_4_NNO_3_, additionally solvated with excess solvents molecules, both TOMANO_3_ and TBP. At high TOMANO_3_ concentrations, the formation and extraction of a four-charge nitrate complex were accompanied by the displacement of TBP molecules from their coordination sphere. The manifestation of a synergistic effect for these compositions of mixtures can be due to the solvation of the three-charge complex (R_4_N)_3_[Nd(NO_3_)_6_] by free TOMANO_3_ and TBP molecules with the formation of the solvate (R_4_N)_3_[Nd(NO_3_)_6_]·R_4_NNO_3_·pTBP, and by solvation by TBP molecules of a four-charge nitrate complex with the formation of the solvate (R_4_N)_4_[Nd(NO_3_)_7_]·pTBP [17].

As implied by the material considered, the main motive for changing the composition of Nd(III) extractable mixed complexes with TOMANO_3_ and TBP is the displacement of phosphoryl ligands from the coordination sphere by nitrate ligands with an increase in the proportion of TOMANO_3_ in the mixture with the formation of more highly charged nitrate complexes in the organic phase. Conversely, with an increase in the concentration of TBP in the mixture, the main motive is to replace the nitrate ligands of TOMANO_3_ with phosphoryl ligands of TBP with a decrease in charge of the nitrate anionic complexes of Nd(III).

According to most of the research cited above on the extraction of REE nitrates by mixtures of quaternary ammonium nitrates and neutral organophosphorus compounds (NOPC) [16,17,18,19,20,21,22,23], the general formula of the resulting compounds can be written as follows: (R_4_N)_n_[Ln(NO_3_)_3+n_·mS], where S is the NOPC molecule, *n* = 1–3, *m* = 1–4. Various studies have shown that the nitrate groups of TOMANO_3_ and phosphoryl groups of NOPC are included in the internal coordination sphere of the mixed complex. The main postulated mechanism of formation of such mixed complexes is the substitution of NOPC molecules in Ln(NO_3_)_3_·3S trisolvates with R_4_NNO_3_ nitrate ligands [8,15,16,18]. The main differences in the compositions of the extractable mixed REE compounds determined in the various studies are in the number of nitrate and phosphoryl groups that are simultaneously part of the complex. As shown above, these discrepancies are primarily due to different concentrations of lanthanide, nitrate QAC, and NOPC when determining the extracted complex.

Another important aspect of REE solvent extraction by mixtures of nitrates, QACs and NOPCs, including TOMANO_3_-TBP, is the possibility of achieving high separation properties against the background of a significant synergistic effect, which leads to an increase in the separation factors of the neighboring REEs. For the REEs of the middle group, TAMANO_3_-TBP mixtures proved to be optimal for separating Sm(III) and Gd(III) with β_Sm(III)/Gd(III)_ ~3–4 [14,19]. This has made it possible to use solutions of TAMANO_3_-TBP mixtures in hydrocarbon diluents to obtain pure Sm_2_O_3_ and Gd_2_O_3_ oxides from concentrates containing samarium, gadolinium, and small quantities of europium on an industrial scale [4,24]. Good results on the SE separation of REEs of the middle group with Aliquat 336 and TBP mixtures using cascades of centrifugal extractors have been achieved in recent years by S.S. Shulin et al. [25,26]. However, the separating properties of the extractant mixtures described above cannot be limited to only the elements of the middle group. The occurrence of a synergistic effect and a wide variety of extracted REE compounds of the light group described above, based on studies [15,16,17], allow us to consider these mixtures as having promise for the separation of La(III), Ce(III), Pr(III), and Nd(III).

The purpose of this research was to determine the possibility of using TOMANO_3_-TBP mixtures for the solvent extraction separation of REEs of the light group from nitrate low-acid solutions to obtain individual compounds of La(III), Ce(III), Pr(III), and Nd(III).

## 2. Results and Discussion

### 2.1. Solvent Extraction Separation of La(III), Ce(III), Pr(III), and Nd(III) Using TOMANO_3_-TBP Mixtures

Experimental data obtained previously on SE of La(III), Ce(III), Pr(III), and Nd(III) from individual weakly acidic nitrate solutions with a salting-out agent was used to evaluate separation factors of REEs depending on the composition of TOMANO_3_-TBP mixtures [16,17]. For the La(III)/Ce(III) pair, La(III) was extracted more effectively into the organic phase, with values β_La(III)/Ce(III)_ ~1.2–1.4 practically throughout the solvent composition change area. For the Ce(III)/Pr(III) pair, Pr(III) was also extracted more effectively into the organic phase, with β_Pr(III)/Ce(III)_ ~1.5–2.3 throughout the solvent composition change area. For the La(III)/Pr(III) pair, extraction should proceed in favor of Pr(III) with β_La(III)/Pr(III)_ ~1.1–1.6 throughout the solvent composition change area. For the Pr(III)/Nd(III) pair, Nd(III) is more effectively extracted, the β_Nd(III)/Pr(III)_ value was 1.3–1.5. Such data estimates enable prediction of the possibility of effective SE separation of light REEs with TOMANO_3_-TBP mixtures for all the pairs if the solvent composition is correctly selected. These mixtures can be used for REE extraction from weakly acidic nitrate media containing not more than 0.2 mol/L HNO_3_, as opposed to TBP, and other NPOEs, extraction of REEs from solutions containing 1–4 mol/L HNO_3_.

When calculated for single-element solutions, β_Ln1/Ln2_ values significantly change on transition to a mixture of light REEs showing that they depend on the REE concentration ratio in the aqueous phase. To determine the composition of TOMANO_3_-TBP mixtures with the most significant differences in distribution ratios, the dependency D_Ln_ during SE with 1.0 mol/L isomolar TOMANO_3_-TBP mixtures in toluene from mixed nitrate solution containing 219 g Ce(III)/L, 106 g La(III)/L, 20 g Pr(III)/L, 55 g Nd(III)/L, and 0.1 mol/L HNO_3_ was studied. This aqueous solution composition was chosen as a model solution that forms after the dissolution of a light REE concentrate separated from a loparite concentrate [3]. Table 2 presents the dependencies of separation factors using 1.0 mol/L isomolar TOMANO_3_-TBP mixtures in toluene.

For the La(III)/Ce(III) pair, the β_La(III)/Ce(III)_ value was >1 (but did not exceed 1.25) in the range of compositions 0.05–0.20 mol/L TOMANO_3_-0.95–0.80 mol/L TBP. In the range of 0.75–0.95 mol/L TOMANO_3_ and 0.25–0.05 mol/L TBP concentrations, inversion in the distribution of La(III) and Ce(III) was observed, and β_Ce(III)/La(III)_ values of 2.5–4 were achieved. This range may be used for separation of Ce(III)/La(III) in the case of distribution of Ce(III) into the organic phase.

Separation for Pr(III)/Nd(III) can be performed over a wide composition range— from 0.15 mol/L TOMANO_3_-0.85 mol/L TBP to 0.85 mol/L TOMANO_3_-0.15 mol/L TBP. The value of β_Pr(III)/Nd(III)_ in this range changed from 2.2 to 4.9, indicating high separation efficacy. For separation of the pair Ce(III)/Pr(III), the composition ranges of 0.15–0.30 mol/L TOMANO_3_-0.85–0.70 mol/L TBP can be selected. In this case, La(III) and Ce(III) will go to the organic phase, while Pr(III) and Nd(III) will stay in the raffinate.

To evaluate the possibility of separation of light REEs including Ce(III)/Pr(III), theoretical calculations were performed for a multistage counter-current SE cascade with scrubbing, according to the methodology suggested by G.V. Korpusov [27]. In cases where the initial solution of REEs with the composition described above is direct to the middle section of the SE cascade (last step of loading), and used a solvent with composition 0.15 mol/L TOMANO_3_-0.85 mol/L TBP in toluene, the number of steps of extraction (loading) of the SE cascade would be ten at an organic to aqueous phase volume ratio (O/A) equal to 18/1, the scrubbing section would have 7–8 steps at O/A equal to 10/1, with the total number of steps equal to 17–18. High O/A ratios are related primarily to low REE distribution ratios that do not exceed 0.1. Such O/A ratio values are not acceptable for industrial SE techniques.

An increase of D_Ln_ in the SE systems considered above can occur through an increase in initial solvent concentration or a change in aqueous phase composition or a rise in total REE concentration, or the introduction of a salting-out agent, for example, NH_4_NO_3_ or other suitable nitrates of an alkali or alkaline-earth metal.

In the case of a concentration increase of the mixture TOMANO_3_/TBP with molar ratio 0.15/0.85 from 1.0 mol/L to 3.3 mol/L in dodecane, the D_Ln_ value increased by an order of magnitude (D_Ce(III)_ = 0.534, D_Pr(III)_ = 0.324), while β_Ce(III)/Pr(III)_ fell to 1.65. Theoretical calculation of a multistage counter-current SE cascade with scrubbing for all newly obtained values of D_Ce(III)_, D_Pr(III)_, and β_Ce(III)/Pr(III)_ allowed the determination that the number of steps in the extraction section should be 32 at O/A equal to 2/1, in the scrubbing section, 24 steps at O/A equal to 4/1, while the total number of cascade steps should be equal to 56.

The addition of a salting-out agent (1.0–2.0 mol/L NH_4_NO_3_) promoted increase in D_Ce(III)_ to 1.1, D_Pr(III)_ to 0.34, and β_Ce(III)/Pr(III)_ to 3.3. In this case, the multistage counter-current SE cascade parameters will be as follows: total steps—24, including 12 steps for the loading section at O/A equal to 1.5/2 and 12 steps for the scrubbing section at O/A equal to 1.5/1.

Thus, the addition of the salting-out agent into weakly acidic solutions containing La(III), Ce(III), Pr(III), and Nd(III) enabled reduction in the total number of steps with a relative decrease in O/A ratio, both in loading and scrubbing sections of the multistage counter-current SE cascade.

### 2.2. Solvent Extraction Separation of La(III), Pr(III), and Nd(III) Using TOMANO_3_-TBP Mixtures

At high concentrations of Ce(III) in the raw feedstock, including in the collective REE concentrate obtained from loparite concentrate, cerium is recovered by a precipitation technique, for example, by oxidation to Ce(IV) and precipitation of hydroxide [3]. Further, to separate the three-component mixture of La(III), Pr(III), and Nd(III), applying the solvent extraction technique using TBP, the first separation is carried out along the La(III)/Pr(III) line and then along the Pr(III)/Nd(III) line to obtain pure La(III), Pr(III), and Nd(III) compounds. According to data in Table 2, for a solution containing all four light REEs, high values of β_La(III)/Pr(III)_ were observed for the range 0.15–0.20 mol/L TOMANO_3_-0.5–0.80 mol/L TBP. In the range of solvent compositions 0.75–0.95 mol/L TOMANO_3_-0.25–0.05 mol/L TBP, Pr(III) was better extracted, while β_Pr(III)/La(III)_ achieved a value equal to 2.2.

When passing to triple-component mixtures formed after recovery of cerium by precipitation, the composition of the solution changed, resulting in changes in the distribution ratios and separation factors. Table 3 presents the values of D_Ln(III)_ and β_Pr(III)/La(III)_ obtained during SE of La(III), Pr(III), and Nd(III) from a model triple-component solution prepared from a collective REE concentrate. This collective REE concentrate was obtained by loparite concentrate reprocessing and cerium removal. The transition from toluene to dodecane was due to practical considerations since hydrocarbon diluents have been more widely applied in practical SE techniques than aromatic ones, including toluene. Experimental data obtained during single contact of aqueous and organic phases showed that D_Pr(III)_ > D_La(III)_, which is in contrast to data in Table 2 for the same range of solvent compositions.

The behavior of La(III) and Pr(III) may be due to changes in the composition of a stock solution with high La(III) concentration, which exceeds by almost five times the initial concentration of Pr(III). At such high La(III) concentrations in the aqueous phase, the organic phase will be close to saturation by La(III), resulting in a significant decrease in D_La(III)_.

For the correct determination of β_La(III)/Pr(III)_ values, the organic phase was mixed with the stock solution three times, thus getting close to solvent saturation with all components of the mixed nitrate solution, as shown in Table 4. TOMANO_3_-TBP mixtures with molar ratio TOMANO_3_/TBP equal to 2/8 or 1/4 in dodecane were used as solvents. In the case when solvent mixture saturation with all solution components was achieved, the D_La(III)_ value exceeded D_Pr(III)_ and D_Nd(III)_. This resulted in the inversion of separation factors for La(III)/Pr(III) in favor of La(III). Moreover, growth of β_La(III)/Pr(III)_ with saturation of the solvent by REEs and expansion of initial total concentration solvents occurred. The maximum β_La(III)/Pr(III)_ value for 2.0 mol/L solution of TOMANO_3_-TBP mixture will reach 1.5, which will lead to growth in the number of steps of the SE cascade.

Effective SE separation of REEs for La(III)/Pr(III) may be achieved up to the value of β_La(III)/Pr(III)_ ≥ 1.6. The saturation of 3.0 mol/L solution of TOMANO_3_-TBP mixture in dodecane can ensure high values for separation factors equal to 1.62–2.43.

Empirical separation factors for La(III)/Pr(III) values calculated for saturation conditions for TOMANO_3_-TBP mixtures in dodecane were used to design the multistage counter-current SE cascade with scrubbing section. Table 5 presents the input data for calculating the multistage counter-current SE cascade using the original PC software “KASKAD”.

**Figure 1:**
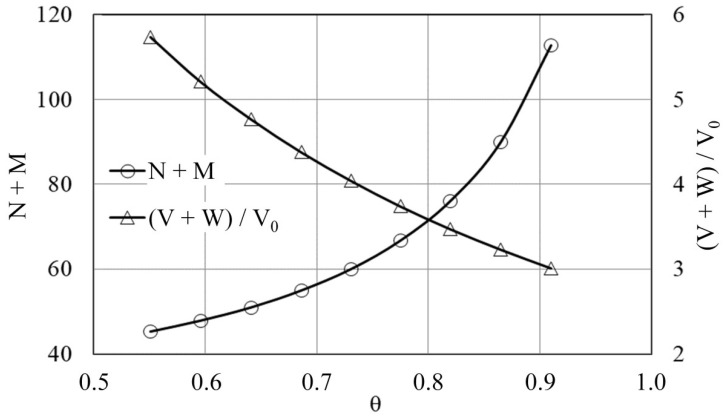
Dependencies (N + M) and ((V + W)/V_0_) from value θ at SE with the mixture 0.4 mol/L TOMANO_3_-1.6 mol/L TBP in dodecane.

Figure 1 provides the results of calculations using the «KASKAD» original PC software in the form of dependencies of the total number of extraction (N) and scrubbing (M) steps (N + M) and parameter ((W + V)/V_0_) from relative selection (θ).

At the intersection of curves in Figure 1, the value θ was 0.795, N + M was 70.25, and (V + W)/V_0_ was 3.64. Based on these calculation values, the following parameters of an operating SE cascade for extraction section were selected: N equal to 40 at O/A equal to 1/1; for the scrubbing section: M equal to 32 at O/A equal to 2/1.

**Figure 2:**
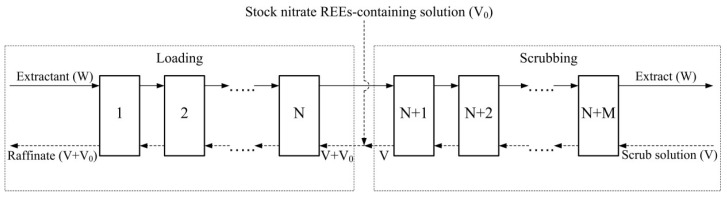
Counter-current extraction cascade operational scheme for separation of REEs to the line La(III)/Pr(III) in nitrate solution containing 258 g La(III)/L, 58 g Pr(III)/L, 141 g Nd(III)/L, and 0.1 mol/L HNO_3_ using 0.6 mol/L TOMANO_3_-2.4 mol/L TBP mixture in dodecane. N—18; M—14; O/A (loading)—2/1; O/A (scrubbing)—2.8/1; W—organic phase flow; V—scrub solution flow; V + V_0_—REE-containing nitrate solution and scrub solution total flow.

Figure 2 shows the operational scheme of the counter-current solvent extraction cascade, which was used to separate the REEs along the line La(III)/Pr(III).

In the case of applying the mixture containing 0.6 mol/L TOMANO_3_-2.4 mol/L TBP in dodecane, for which β_La(III)/Pr(III)_ was 2.43, similar calculations were carried out. The optimal values of relative selection at the intersection of curves (N + M) = *f*(θ) and (V + W)/V_0_ = *f*(θ) was 0.815, N + M was 30.5, and (V + W)/V_0_ was 1.4. Based on the calculated values, the following parameters of an operating SE cascade were selected: for extraction (loading) section, N equal to 18 at O/A equal to 2/1, and for the scrubbing section, M equal to 14 at O/A equal to 2.8/1.

Thus, an increase in separation factors for the pair La(III)/Pr(III) for SE with mixture 0.6 mol/L TOMANO_3_-2.4 mol/L TBP in dodecane enabled reduction in the number of steps in the multistage counter-current SE cascade from 72 to 32 with an increase of O/A for the extraction and scrubbing sections by 2 and 1.4 times, respectively.

It should be noted that, in the initial stage of research, the experimental results obtained earlier [16,17] were used to develop a counter-current multistage solvent extraction process for the separation of La(III), Ce(III), Nd(III), and Pr(III). These data made it possible to determine the synergic compositions of solvent mixtures, to establish the main characteristics of the separation process of La(III), Ce(III), Nd(III), Pr(III), and the modification of the operational scheme of the solvent extraction cascade. The results of the study presented in this article made it possible to fully substantiate the previously obtained data and optimize the separation process using a calculated and mounted multistage solvent extraction laboratory unit. The results obtained are important for developing and optimizing new solvent extraction selective systems for REE separation in weakly acidic nitrate media.

## 3. Materials and Methods

For this study, the oxides, La_2_O_3_, Pr_6_O_11_, Nd_2_O_3_, and compounds Ce(NO_3_)_3_·6H_2_O, HNO_3_, NH_4_OH, Trilon B (2-aqueous disodium ethylene diamine-*N*,*N*,*N*′,*N*′-tetraacetic acid), xylenol orange, sodium salt of diphenylamine-4-sulphonic acid, and chemically pure grade AgNO_3_ were used.

**Figure 3:**
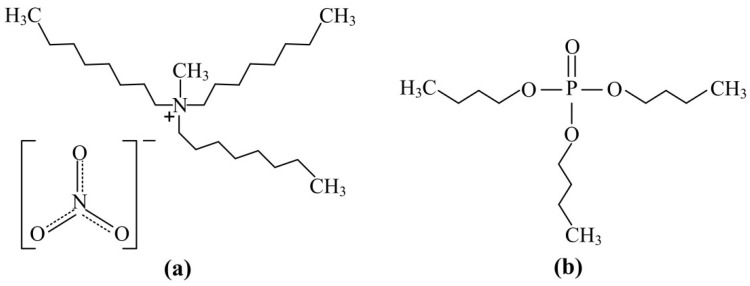
Structure of methyltri-n-octylammonium nitrate (**a**) and tri-n-butyl phosphate (**b**).

As solvents, methyltri-n-octylammonium (MTOA) nitrate and pure grade tri-n-butyl phosphate were used (see Figure 3). MTOA nitrate was synthesized as per original techniques from MTOA methylsulphate (product of tri-n-octylamine alkylation with dimethylsulphate) with 99.9% content of CH_3_(C_8_H_17_)_3_NNO_3_ (in terms of dry product). Toluene and dodecane of chemically pure grade were used as diluents. By dissolving a precisely weighed relevant oxide in the estimated quantity of concentrated HNO_3_ solution stock individual nitrate solutions of La(III), Pr(III), and Nd(III) were prepared. After completely dissolving the REE oxides, ammonium nitrate was added to the solution. To the resulting solution, a calculated and exactly weighed quantity of solid NH_4_NO_3_ was added and dissolved. The pH of the solution was adjusted to 2 (by addition of 1.0 mol/L NH_4_OH solution), corresponding to an HNO_3_ concentration equal to 0.01 mol/L. For the preparation of Ce(III)-containing nitrate solution, a precisely weighed quantity of crystalline Ce(NO_3_)_3_·6H_2_O was dissolved in an aqueous solution containing 4.0 mol/L NH_4_NO_3_ with pH ~2 (correction using HNO_3_ solution).

By dissolving precisely weighed quantities of REE oxides in the estimated amount of concentrated HNO_3_ solution stock mixed nitrate solutions of La(III), Pr(III), and Nd(III) were prepared. After complete dissolution of the REE oxides, an exactly weighed amount of crystalline Ce(NO_3_)_3_·6H_2_O was added to the solution. After Ce(NO_3_)_3_·6H_2_O dissolution, the content of HNO_3_ in nitrate solution was adjusted to 0.1–0.2 mol/L by adding an estimated quantity of 1.0 mol/L HNO_3_ or by neutralizing of excessive acid with 1.0 mol/L NH_4_OH solution.

Organic solutions of the solvent mixtures were prepared by dissolving precisely weighed quantities of 1.96 mol/L TOMANO_3_ and 3.65 mol/L TBP in toluene or dodecane in a 50 mL or 100 mL volumetric flask. Estimated quantities of 3.65 mol/L (100%) TBP and 1.96 mol/L (100%) MTOA nitrate were put into the flask, following which the mixture was diluted with the diluent to the required volume. The content of TOMANO_3_ in mixed solutions was determined by potentiometric titration of the iodide form of the quaternary salt with 0.1 mol/L aqueous solutions of AgNO_3_ in the presence of an iodide-selective electrode (Radelkis, Hungary), in 50% *v*/*v* n-propanol + 50% *v*/*v* aqueous medium.

Solvent extraction from individual REE solutions was performed in 10–25 mL glass-stoppered tubes at room temperature 20 ± 2 °C, while intensively stirring at O/A equal to 1/1 (5–10 mL per phase). The phase contact time was 15 min and the phase separation time was 10–20 min. After which a sample of aqueous phase aliquot was taken and tested for metal content using the Trilon B titration method in the presence of xylenol orange [28].

Solvent extraction from mixed REE solutions was performed in 250 mL separating glass funnels equipped with mechanic stirrers at 20 ± 2 °C at required O/A. The phase contact time was 15 min and the phase separation time was 15–20 min. On phase disengagement, the aqueous phase was separated from the organic phase and tested for content of the total of elements La(III), Ce(III), Pr(III), and Nd(III) with the Trilon B titration method in the presence of xylenol orange [28]. The content of Pr(III) and Nd(III) in mixed solutions was determined spectrophotometrically based on absorption of individual bands in the electronic spectrum, at 444 nm for Pr(III) and at 794 nm for Nd(III). The concentration of Ce(III) in mixed solutions was determined by redox titration using ferrous ammonium sulfate in the presence of the sodium salt of diphenylamine-4-sulphonic acid as an indicator [29]. The concentration of La(III) in mixed solutions was calculated by the difference between the total concentration of all the four metals and the concentration of Ce(III), Pr(III), and Nd(III).

The content of La(III), Ce(III), Pr(III), and Nd(III) in phases at equilibrium was calculated from differences in concentrations in the stock and aqueous solutions at equilibrium. In the event of multiple contacts of mixed solutions with the organic phase, the concentration of metals in the organic phase was determined in aqueous phase after double stripping with 4.0 mol/L aqueous solution of HNO_3_ at O/A equal to 1/1, using the methods described above. For verification and confirmation of the obtained experimental data, a comparison was carried out between the calculated and experimentally determined concentrations of each REE.

Distribution ratios of Ln (D_Ln_) were calculated by the formula:

D_Ln_ = C_ORG_/C_AQ_ = (C_INIT_ − C_AQ_)/C_AQ_ = C_INIT_/C_AQ_ − 1 (1)

where C_INIT_, C_AQ_, and C_ORG_ are initial concentrations of Ln in initial aqueous nitrate solution, aqueous and organic phases after extraction, respectively.

Synergistic effect (factor) S_Ln_ was calculated by the formula:

S_Ln_ = D_exp_/(D_1_ + D_2_) (2)

where D_exp_ is the experimentally determined distribution ratio of Ln for the solution of solvent mixture and D_1_ and D_2_ are the distribution ratios of Ln for an individual solution of each solvent at their equal concentrations in the mixed solution. At S > 1, a synergistic effect is observed, while at S < 1, an antagonistic effect is observed [15,17].

Calculations of multistage counter-current SE cascades containing the loading and scrubbing section during delivery of stock nitrate REE-containing solution into the middle cascade section (the last stage of loading section) were performed using KASKAD original PC software. The KASKAD program was developed according to the theoretical methodology by G.V. Korpusov [27]. Calculation of cascade steps and O/A ratio in the loading and scrubbing section of the SE cascade was performed by the equations binding these parameters with the recovery parameters of the extractable components, firstly, with the relative selection (θ), enrichment level (Q), and separation factor (β_Ln1/Ln2_). Enrichment level (Q) is equal to change in the relative concentration of a component. Relative selection (θ) value is calculated according to the following formula:

θ = θ′/θ′_max_ (3)

where θ′ (selection) is defined as the proportion of the pure component extracted into the raffinate or extract in relation to the total amount of this component feeding to the extraction or scrubbing, respectively. The maximum possible selection value (θ′_max_) is assumed to be equal to (β_Ln1/Ln2_ − 1)/β_Ln1/Ln2_ (when obtaining a purified individual REE compound) [27].

Then the dependencies of the total number of loading (N) and scrubbing (M) steps (N + M) and the ratio (W + V)/V_0_ are plotted, where W, V, and V_0_ are flows of the organic phase, scrubbing solution, and REEs-containing nitrate solution, respectively. The optimal value of relative selection is determined at the intersection point of the curves (N + M) = *f*(θ) and (V + W)/V_0_ = *f*(θ). The optimal value of relative selection is used to calculate the optimal number of steps and O/A ratio in each of the SE cascade sections.

## 4. Conclusions

Solvent extraction of light REEs, i.e., La(III), Ce(III), Pr(III), and Nd(III) using methyltri-n-octylammonium nitrate and tri-n-butyl phosphate mixtures from weakly acidic nitrate solutions is due to the formation of several mixed complexes. The composition of REE-extractable complexes depends on the molar ratios of REE nitrate, TOMANO_3_, and TBP, which are components of the mixed complex. Differences in the composition of the complexes of each element with the same composition of solvent mixture enables separation of light REEs with high separation factors. An increase in distribution ratios in such systems is due to a rise in solvent concentration in the mixture at the same molar ratio. This has a positive effect on solving a practical issue of the separation process, resulting in a lowered number of steps in a multistage counter-current solvent extraction cascade and O/A ratio both in the extraction and scrubbing sections. The TOMANO_3_-TBP organic mixtures are proposed for separation of light REEs because of their increased extractability due to synergic properties. This makes it possible to extract REEs from aqueous solutions containing no more than 0.2 mol/L of nitric acid and to achieve high separation factors for neighboring elements. The results obtained confirm the possibility of applying such synergic mixtures for practical industrial solvent extraction separation of La(III), Ce(III), Pr(III), and Nd(III).

## Figures and Tables

**Table 1:** Separation of rare-earth elements from aqueous nitrate solutions by solvent extraction techniques.

REE	Aqueous Solution Composition	Extractant Mixture Diluent	Synergic Complex	Method for Determination	Ref.
La(III)	NH_4_NO_3_ (3.0 mol/L) pH = 1.5–2	Adogen 464:TBP = 1:1 (0.8 mol/L) m-xylol	R_4_N[La(NO_3_)_4_·TBP]	Isomolar series	[6]
La(III)	Nitrate solutions	Nitrate Adogen 464-DIOMP (1.0 mol/L) toluene	R_4_N[La(NO_3_)_4_·DIOMP]	Isomolar series; Slop analysis	[7]
Ce(III)	HNO_3_ (3.0 mol/L)	TLMANO_3_-TBP toluene	(R_4_N)_2_[Ce(NO_3_)_5_·TBP]	Slop analysis	[8]
Pr(III)	Nitrate solutions	Aliquat 336-TBP benzene and CCl_4_	Pr(NO_3_)_3_·2R_4_NNO_3_·TBP or (R_4_N)_2_[Pr(NO_3_)_5_·TBP]		[9,10]
Nd(III)	NH_4_NO_3_ (3.0 mol/L) Nd(NO_3_)_3_ (0.17 mol/L)	BTA nitrate-TBP m-xylol	(R_4_N)[Nd(NO_3_)_4_·TBP] (R_4_N(R_4_NNO_3_)*_p_*_–1_)·[Nd(NO_3_)_4_·TBP]		[11]
La(III) Ce(III) Pr(III) Nd(III)	Nitrate solutions, pH = 2	TAMANO_3_-TBP	(R_4_N)_2_[Ln(NO_3_)_5_·2TBP]	Organic phase saturation; Mathematical modeling	[12,13]
Nd(III)	NH_4_NO_3_ (8.0 mol/L) Nd(NO_3_)_3_ (5·10^–4^ mol/L) pH = 2.6	TAMANO_3_-TBP TAMANO_3_-TiBP TAMANO_3_-TsBP	R_4_N[Nd(NO_3_)_4_·2TBP] R_4_N[Nd(NO_3_)_4_·(4–5)TiBP]	Isomolar series	[14]

**Table 2:** Dependence of separation factors for light REEs using 1.0 mol/L isomolar TOMANO_3_-TBP mixtures in toluene.

[TOMANO_3_]/[TBP], mol/L/mol/L	β_La(III)/Ce(III)_	β_Ce(III)/Pr(III)_	β_Pr(III)/Nd(III)_	β_Ce(III)/La(III)_	β_La(III)/Pr(III)_	β_Pr(III)/La(III)_
0.05/0.95	1.09	1.30	2.74	0.92	1.42	0.71
0.10/0.90	0.90	1.59	1.16	1.11	1.43	0.70
0.15/0.85	1.15	1.71	2.58	0.87	1.97	0.51
0.20/0.80	1.25	1.53	4.41	0.80	1.91	0.52
0.25/0.75	0.25	1.41	4.91	4.03	0.35	2.87
0.30/0.70	1.02	1.73	3.07	0.98	1.77	0.57
0.35/0.65	0.67	1.37	1.97	1.50	0.91	1.10
0.40/0.60	0.31	1.70	3.66	3.25	0.52	1.92
0.45/0.55	0.85	1.39	2.83	1.17	1.18	0.84
0.50/0.50	1.04	1.53	2.20	0.96	1.59	0.63
0.55/0.45	0.74	1.85	4.28	1.34	1.38	0.72
0.60/0.40	1.19	1.42	2.74	0.84	1.68	0.59
0.65/0.35	1.06	1.67	2.30	0.94	1.77	0.57
0.70/0.30	1.11	1.59	1.65	0.90	1.77	0.57
0.75/0.25	0.30	1.79	2.26	3.36	0.53	1.88
0.80/0.20	0.39	1.54	2.92	2.55	0.61	1.65
0.85/0.15	0.33	1.47	2.89	3.06	0.48	2.08
0.90/0.10	0.39	1.65	1.17	2.56	0.64	1.55
0.95/0.05	0.25	1.83	1.44	4.02	0.46	2.19

[TOMANO_3_], [TBP]—the concentration of TOMANO_3_ and TBP in the organic mixture.

**Table 3:** Values D_La(III)_, D_Pr(III)_, and β_La(III)/Pr(III)_ during SE from the solution containing 240 g La(III)/L, 49 g Pr(III)/L, 145 g Nd(III)/L, and 0.2 mol/L HNO_3_ using 1.0 mol/L isomolar TOMANO_3_-TBP mixtures in dodecane. One extraction step, O/A equal to 1/1.

[TOMANO_3_]/[TBP], mol/L/mol/L	Molar Ratio TOMANO_3_/TBP	D_Pr(III)_	D_La(III)_	β_Pr(III)/La(III)_
0.2/0.8	2/8	0.11	0.09	1.25
0.4/1.6	2/8	0.24	0.12	2.07
0.6/2.4	2/8	0.36	0.17	2.12
0.7/2.8	2/8	0.44	0.14	3.09
0.1/0.9	1/9	0.10	0.12	1.15
0.2/1.8	1/9	0.18	0.17	1.06
0.3/1.7	1/9	0.31	0.14	2.15
0.35/3.15	1/9	0.34	0.20	1.71

[TOMANO_3_], [TBP]—the concentration of TOMANO_3_ and TBP in the organic mixture.

**Table 4:** Values of D_Ln_ and β_Ln1/Ln2_ during SE from a solution containing 258 g La(III)/L, 58 g Pr(III)/L, 141 g Nd(III)/L, and 0.1 mol/L HNO_3_ using TOMANO_3_-TBP mixtures in dodecane.

Step Number	D_La(III)_	D_Pr(III)_	D_Nd(III)_	β_La(III)/Pr(III)_	β_La(III)/Nd(III)_	β_Pr(III)/Nd(III)_
0.4 mol/L TOMANO_3_-1.6 mol/L TBP in dodecane						
1	0.497	0.631	0.359	0.79	1.38	1.76
2	0.830	0.659	0.361	1.26	2.30	1.83
3	0.977	0.650	0.367	1.50	2.66	1.77
0.6 mol/L TOMANO_3_-2.4 mol/L TBP in dodecane						
1	0.830	0.629	0.548	1.32	1.51	1.15
2	1.151	0.708	0.497	1.62	2.32	1.42
3	1.688	0.695	0.504	2.43	3.35	1.38

**Table 5:** Input data for calculation of multistage counter-current solvent extraction separation of La(III) and Pr(III) using «KASKAD» original PC software.

Parameter	Value
The distribution ratio value of the low extractable component (D_Pr(III)_)	0.650
The distribution ratio value of the high extractable component (D_La(III)_)	0.977
Value of separation factor (β_La(III)/Pr(III)_)	1.50
Minimum relative selection, θ_min_	0.551
Enrichment level (Q), %	99.9
The relative flow rate of the extractable component mixture (V_0_), L/min	1.0
Total points for calculation	10
Precision of calculation	0.1

## Data Availability

Data are presented in the paper and available from the authors.

## Sample Availability

Samples from the compounds are available from the authors.

## References

[1] Mikhailov V.A. (2010). Redkozemel’nye Rudy Mira: Geologiya, Resursy, Ekonomika (Rare Earth Ores of the World: Geology, Resources, Economics).

[2] Polyakov E.G., Nechaev A.V., Smirnov A.V. (2018). Metallurgiya Redkozemel’nyh Metallov (Metallurgy of Rare Earth Metals).

[3] Stepanov S.I., Chekmarev A.M. (2016). Razdelenie Redkozemel’nyh Elementov (Separation of Rare Earth Elements).

[4] Chuprinko V.G., Ohapkin O.G., Ozerova L.S., Stepanov G.A., Kosynkin V.D., Selivanovsky A.K., Fedulova T.T., Makarov V.I., Arzhatkina L.A. (1995). Sposob Razdeleniya Ittriya i Redkozemel’nyh Elementov (Method of Separation of Yttrium and Rare Earth Elements). Patent RU.

[5] Valkov A.V., Sergievsky V.V. (2011). Smesi fosfororganicheskih ekstragentov s nitratom metiltrialkilammoniya v tekhnologii samariya, gadoliniya, terbiya i erbiya (Mixtures of organophosphorus extractants with methyltrialkylammonium nitrate in the technology of samarium, gadolinium, terbium and erbium). Proceedings of the Prospects for the Extraction, Production and use of REM, Theses and Reports of the 1st Russian Scientific and Practical Conference.

[6] Mikhailichenko A.I., Goryacheva E.G., Aksenova N.M., Denisov A.F. (1982). Ekstrakciya lantana i aktiniya smes’yu nitrata trialkilmetilammoniya i tributilfosfata (Lanthanum and actinium extraction by mixture of trialkylmethylammonium nitrate and tributyl phosphate). Radiokhimiya.

[7] Goryacheva E.G., Vdovina L.V., Miuskova N.M. (1984). Ekstrakciya redkozemel’nyh metallov smesyami nitrata trialkilmetilammoniya i diizooktilmetilfosfonata iz nitratnyh rastvorov (Extraction of rare earth metals with mixtures of trialkylmethylammonium nitrate and diisoctylmethylphosphonate from nitrate solutions). Sci. Work. GIREDMET Res. Field Rare Earth Met. Prod. Electron. Spec. Opt..

[8] Kolaric Z., Puzic R.G., Maksimovic Z.B. (1969). Solvent extraction of some metals by mixtures of tributylphosphate with alkyl ammonium nitrates. J. Inorg. Nucl. Chem..

[9] Dukov I., Kassabov G., Genov L. (1979). Synergic extraction of praseodymium by mixtures of tributylphosphate and quaternary ammonium salt. Monatch. Chem..

[10] Dukov I., Kasabov G., Genov L. (1980). Sinergetna ekstrakciya na prazeodim smesi ot tributilfosfat i chetvertichna amonieva sol (Synergetic extraction of praseodymium with a mixture of tributyl phosphate and the quaternary amonium salt). Godishnik Na visshij Himiko-Tekhnologich. Inst..

[11] Mikhlin E.B., Rozen A.M., Norina T.M., Nikonov V.N., Afonina T.A., Tumanov A.V. (1977). Ekstraktsiya redkozemel’nykh elementov smesyami soli chetvertichnykh ammonievykh osnovanii i tributilfosfata iz nitratnykh rastvorov (Extraction of rare-earth elements with ternary ammonium base salts and tributylphosphate from nitrate solutions). Radiokhimiya.

[12] Kopyrin A.A., Puzikov E.A., Pyartman A.K. (1994). Zakonomernosti ekstrakcii nitratov lantanidov i ittriya smesyami ekstragentov na osnove nitrata trialkilmetilammoniya i nejtral’nyh fosfororganicheskih soedinenij (Patterns of extraction of lanthanide nitrates and yttrium with mixtures of extractants based on trialkylmethylammonium nitrate and neutral organophosphorus compounds). Proceedings of the Abstracts of Reports, 1st Russian Radiochemistry Conference.

[13] Pyartman A.K., Kopyrin A.A., Kovalev S.V., Keskinov V.A. (1996). Vliyanie razbavitelej na processy ekstrakcii nitratov lantanidov(III) i ittriya(III) nitratami chetvertichnyh ammonievyh osnovanij i tri-n-butilfosfatom (Influence of diluents on the processes of extraction of lanthanide nitrates(III) and yttrium(III) with nitrates of quaternary ammonium bases and tri-n-butyl phosphate). Proceedings of the Abstracts of Reports of the 2nd International Symposium, Problems of Integrated ore Use.

[14] Valkov A.V., Khmelevskaya N.D. (2018). Ekstrakciya redkozemel’nyh elementov smesyami izomerov tributilfosfata s nitratom trialkilmetilammoniya (Extraction of rare earth elements with mixtures of isomers of tri-n-butyl phosphate with trialkylmethylammonium nitrate). News High. Educ. Institutions. Chem. Chem. Technol..

[15] Stepanov S.I., Chekmarev A.M. (2004). Ekstrakciya Redkih Metallov Solyami Chetvertichnyh Ammonievyh Osnovanij (Extraction of Rare Metals with Salts of Quaternary Ammonium Bases).

[16] Stepanov S.I., Hoa N.T., Chekmarev A.M., Tsivadze A.Y. (2021). Chemistry of the extraction of La(NO_3_)_3_ and Ce(NO_3_)_3_ from nitrate solutions with mixtures of tri-n-octylmethylammonium nitrate and tri-n-butyl phosphate in toluene. Theor. Found. Chem. Eng..

[17] Stepanov S.I., Hoa N.T., Chekmarev A.M., Tsivadze A.Y. (2021). Chemistry of solvent extraction of Nd(NO_3_)_3_ and Pr(NO_3_)_3_ from nitrate solutions with TOMAN–TBP mixtures in toluene. Dokl. Chem..

[18] Huang C.-H., Bautista R.G. (1983). The synergistic extraction of Sm(NO_3_)_3_ and Gd(NO_3_)_3_ by a mixture of TBP and Aliquat 336 in AMSCO and ammonium nitrate medium. Sep. Sci. Technol..

[19] Dian C., Sengchung L., Roufei Z. (1983). Separation and preparation of gadolinium of high purity (99.99%) by synergic extraction with quaternary ammonium salt and TBP or di(1-metyl-heptyl)metylphosphonate mixed extractants. Proceedings of the International Solvent Extraction Conference, AICHE ISEC ‘83.

[20] Mikhailichenko A.I., Karmannikov V.P., Klimenko M.A., Fedulova T.V. (1984). Nekotorye zakonomernosti raspredeleniya redkozemel’nyh metallov pri ekstrakcii smesyami nitrata trialkilbenzilammoniya i diizooktilmetilfosfonata (Some patterns of the distribution of rare earth metals during extraction with mixtures of trialkylbenzylammonium nitrate and diisoctylmethylphosphonate). Sci. Work. GIREDMET Res. Field Rare Earth Met. Prod. Electron. Spec. Opt..

[21] Mikhlin E.B., Rosen A.M., Norina T.M., Nikonov V.N., Afonina T.A. (1976). Ekstrakciya redkozemel’nyh elementov smesyami nejtral’nyh ekstragentov iz nitratnyh rastvorov (Extraction of rare-earth elements with mixtures of neutral extractants from nitrate solutions). Russ. J. Inorg. Chem..

[22] Pyartman A.K., Kopyrin A.A., Kovalev S.V., Keskinov V.A., Bogatov K.B. Vliyanie razbavitelej na processy ekstrakcii nitratov lantanidov(III) i ittriya(III) nitratami chetvertichnyh ammonievyh osnovanij i tri-n-butilfosfatom (Effect of diluents on the extraction processes of lanthanide(III) and yttrium(III) nitrates of quaternary ammonium bases and tri-n-butyl phosphate). Proceedings of the Abstracts of Reports International Conference, Rare Earth Metals: Raw Material Processing, Technology of Compounds and Related Products.

[23] Kopyrin A.A., Pyartman A.K. Zakonomernosti raspredeleniya lantanidov(III) v mnogokomponentnyh sistemah pri ispol’zovanii ekstragentov razlichnyh klassov i ih smesej (Patterns of lanthanide (III) distribution in multicomponent systems using extractants of various classes and their mixtures). Proceedings of the Abstracts of Reports 11th Russian Conference on Extraction.

[24] Duyckaerts G., Desreux J.F., Lucas B.H., Ritcey G.M., Smith H.W. (1979). Resent developments and new combinations of extractants in synergic processes. Proceedings of the International Solvent Extraction Conference ISEC 77.

[25] Shulin S.S., Galieva G.N., Chighevskaya S.V., Pletuhina J.V., Saveliev N.S. (2018). Extraction separation of rare earth elements of the medium group with isomolar mixtures of Aliquat^®^ 336–TBP and Cyanex^®^ 572–TBP from nitric solutions. Inorg. Mater..

[26] Shulin S.S., Abramov A.M., Galieva J.N., Sobol Y.B., Kulagin B.R. (2015). Issledovanie po ekstrakcionnomu razdeleniyu koncentratov redkozemel’nyh elementov «srednetyazheloj» gruppy razlichnymi ekstragentami (Study on the extraction separation of concentrates of rare-earth elements of the “medium-heavy” group by various extractants). Non-Ferr. Metall..

[27] Sinitsyn N.M., Corpusov G.V., Zaitsev L.M., Levin V.I., Sinitsyna S.M., Prokopchuk Y.Z., Zaitsev B.A., Grivkova A.I., Voloshenko L.L., Nikolaev A.N. (1970). Redkozemel’nye elementy (Rare earth elements). Himiya Dolgozhivushchih Oskolochnyh Elementov (Chemistry of Long-Lived Fission Elements).

[28] Busev A.I., Tiptsova V.G., Ivanov V.M. (1978). Rukovodstvo po Analiticheskoj Himii Redkih Elementov (Manual on Analytical Chemistry of Rare Elements).

[29] Ryabchikov D.I., Ryabukhin V.A. (1966). Analiticheskaya Himiya Redkozemel’nyh Elementov i Ittriya (Analytical Chemistry of Rare Earth Elements and Yttrium).

